# Modeling viscoelasticity through spring–dashpot models in intermittent-contact atomic force microscopy

**DOI:** 10.3762/bjnano.5.224

**Published:** 2014-11-18

**Authors:** Enrique A López-Guerra, Santiago D Solares

**Affiliations:** 1 Department of Mechanical Engineering, University of Maryland, College Park, Maryland 20742, United States; Current Address: Department of Mechanical and Aerospace Engineering, George Washington University, Washington, DC 20052, United States

**Keywords:** atomic force microscopy, creep, dissipated energy, multifrequency, stress relaxation, tapping mode, viscoelasticity

## Abstract

We examine different approaches to model viscoelasticity within atomic force microscopy (AFM) simulation. Our study ranges from very simple linear spring–dashpot models to more sophisticated nonlinear systems that are able to reproduce fundamental properties of viscoelastic surfaces, including creep, stress relaxation and the presence of multiple relaxation times. Some of the models examined have been previously used in AFM simulation, but their applicability to different situations has not yet been examined in detail. The behavior of each model is analyzed here in terms of force–distance curves, dissipated energy and any inherent unphysical artifacts. We focus in this paper on single-eigenmode tip–sample impacts, but the models and results can also be useful in the context of multifrequency AFM, in which the tip trajectories are very complex and there is a wider range of sample deformation frequencies (descriptions of tip–sample model behaviors in the context of multifrequency AFM require detailed studies and are beyond the scope of this work).

## Introduction

Atomic force microscopy (AFM) has evolved rapidly since its invention in the mid-1980s [1] and has been used since then for measuring topography and probe–sample forces on micro- and nanoscale surfaces in different environments. Tapping mode AFM (amplitude modulation, AM-AFM) is the most common dynamic method and has been the subject of thorough studies [2–6]. In tapping mode AFM damage or wear of the tip and surface are reduced with respect to contact-mode AFM due to lower friction and lateral forces, which makes it more applicable for imaging soft samples, such as polymers and biological surfaces. Tapping mode AFM has the additional advantage that it records a phase contrast simultaneously with the acquisition of topography, which can be very useful in the study of heterogeneous samples [7–10]. Moreover, the observables in tapping mode AFM (phase and amplitude) can provide quantitative information about the dissipative and conservative tip–sample interactions by converting them to energy-based quantities, namely the dissipated power (*P*_ts_) and virial (*V*_ts_) [9,11].

Although several authors have achieved quantification of energy dissipation processes [12–15], the further utilization of that information to derive material properties is not trivial in tapping mode AFM. The nature of the technique with its intermittent contact, during which the probe interacts with nonlinear tip–sample forces ranging from attractive to repulsive, hinders the derivation of simple relationships between observables and sample properties. Furthermore, the extraction of sample properties becomes especially challenging when studying viscoelastic materials. Despite the obstacles, analytical and numerical simulations have been performed as an attempt to estimate quantities such the sample loss tangent (a common term used in the characterization of viscoelastic samples) [16] although it has been reported that this approach can be inaccurate for intermittent-contact applications [17]. Notably, one of the key factors preventing the extraction of reliable material information has been the absence of physically accurate models for viscoelastic samples. On the other hand, better quantitative agreement has been accomplished through contact-mode based techniques such as contact resonance AFM (CR-AFM) [17], band excitation AFM (BE-AFM) [18–19] and dual-amplitude resonance tracking AFM (DART-AFM) [20]. These techniques operate in a regime of quasi-linear tip–sample forces by using very small cantilever oscillation amplitudes, but as a result only provide linear viscoelasticity information and characterization can be slow for CR-AFM and BE-AFM due to the pixel-based measurement procedures used.

Significant progress has been recently achieved with regards to fast and simultaneous topographical and spectroscopic characterization of viscoelastic materials through the use of multifrequency AFM [21]. This work represents an important milestone in rapid and quantitative multi-property characterization, although it has so far only been realized in the context of a very simple viscoelastic model that is generally not physically accurate (this model is discussed in detail below). In fact, most of the current models used in AFM simulation do not take into account fundamental viscoelastic behaviors, such as stress relaxation, creep or multiple relaxation times, which are very distinct features in materials that exhibit rate-dependent behaviors, such as polymers [22]. A recent attempt has been made to model viscoelastic samples in AFM by using a standard linear solid (SLS) model (which is also discussed below) in order to include basic rate dependent properties [22–25]. Although this is a reasonable step, further sophistication is still required in order to realistically capture the nonlinear rate-dependent behaviors.

The present paper explores the nature and behavior of spring–dashpot sets as examples of models that can be used for representing viscoelastic surfaces. The first part of the study reviews the simplest models used in the context of linear viscoelasticity within AFM, followed by a discussion of more sophisticated spring–dashpot models. The second part of the study evaluates in detail the force–distance curve and dissipation behavior of these models, focusing on single-eigenmode tip–sample impacts. Throughout the paper, the advantages and disadvantages of the various models are discussed, along with possible enhancements that can lead to more accurate simulation of viscoelastic material characterization with AFM.

## Results and Discussion

### Model descriptions

#### Linear Maxwell model

**Figure 1:**
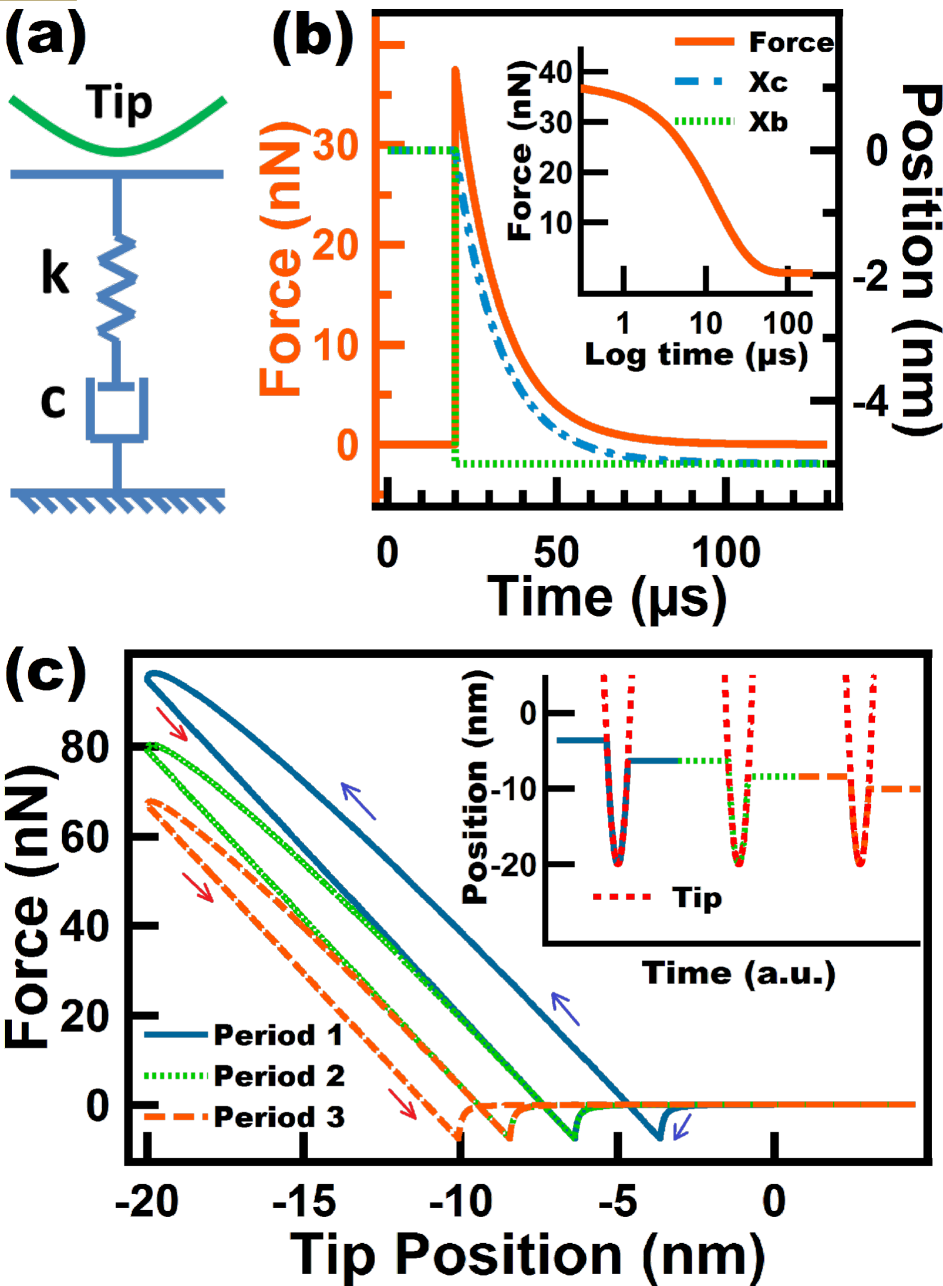
(a) Linear Maxwell model schematic; (b) stress relaxation simulation performed on a Linear Maxwell surface. The surface position (*X*_b_) is depressed to a constant position of 5 nm below its unperturbed state, starting at *t* = 20 µs. The inset shows the same stress relaxation experiment but starting at *t* = 0 µs, and the horizontal axis is intentionally plotted by using a logarithmic scale to show the inflection point corresponding to its single relaxation time. (c) Force–distance tip trajectories (the trajectory proceeds in the counterclockwise direction) for a prescribed sinusoidal tip trajectory given by *z*(*t*) = 80 nm + (100 nm) sin(ω*t*), where ω is 2π times 25 kHz. The inset shows three consecutive tip–sample taps, each one separated by a fundamental period equal to 1/25 ms. The Linear Maxwell parameters used were *k* = 7.5 N/m and *c* = 1.0 × 10^−4^ N·s/m. The blue and red arrows in (c) correspond to approach and retraction of the tip, respectively.

The Linear Maxwell model is one of the simplest spring–dashpot sets. It consists of a spring arranged in series with a dashpot (Figure 1a). This model is known for successfully describing stress relaxation (time-dependent drop in stress under a constant strain) and for failing to describe creep (time-dependent strain relaxation under a constant stress). The latter precludes the existence of a mechanism for surface recovery upon deformation. As a consequence, the sample continuously yields to lower positions when impacted by the AFM tip, such that in subsequent impacts the tip meets the sample at lower and lower heights (see inset of Figure 1c). This also means that a tapping tip would not be able to reach steady state as the surface is continuously yielding (i.e., the probe would reach steady state only when the Linear Maxwell sample has yielded sufficiently to allow the tip to oscillate at its free oscillation amplitude, without any tip–sample interaction). Since we are interested in the response of the Linear Maxwell sample with an intermittent contact probe, we have used a prescribed tip trajectory for the simulations in Figure 1. We have thus prescribed the tip motion as *z*(*t*) = *z*_c_ *+ A*·sin(ω*t*) while allowing surface relaxation. In this case the tip was forced to travel down to 20 nm below the original surface position for each tap, as shown in the inset of Figure 1c. The inset also shows how the surface yields for each consecutive tap, and it can also be seen that it experiences only a partial recovery without returning to its original position. When the tip goes down, the Linear Maxwell surface partially relaxes through the dashpot, which is the element that causes relaxation of the force stored in the spring. During retraction the sample experiences an elastic recovery that is proportional to the force stored in the spring, which could not fully relax during the approach. However, the sample does not experience viscous recovery because the dashpot does not have a mechanism to travel back up and return to its original position.

Despite the limitations of the Linear Maxwell model, it is able to model dissipation which is evidenced by the presence of a hysteresis loop in the force–distance (FD) curve (see Figure 1c). This dissipation loop arises from the gap between the energy input (energy given by the cantilever to the surface during approach) and the energy output (energy returned by the surface to the cantilever during retract). In spring–dashpot models this gap is caused by the relief of some of the stress accumulated in the springs through the dashpots. Another advantage of the Linear Maxwell model is that it gives a qualitatively accurate description of a FD curve for a viscoelastic sample during a single impact. Figure 1c shows FD curves containing two minima that arise from the fact that the tip encounters and leaves the sample at different heights (the surface remains depressed when the tip leaves the sample). The lack of surface recovery of the Linear Maxwell surface is also evidenced in the force–distance curves of consecutive taps where it can be seen that each loop is shifted to the left where the retract point of a previous tap is the approach point of the subsequent oscillation (see Figure 1c). It is also worth mentioning that all our simulations include long range attractive forces, incorporated through the Hammaker equation (see details in the Methods section) in order to obtain results that are more directly applicable to AFM.

Figure 1b shows a stress relaxation experiment for a Linear Maxwell arm. It can be seen that as time increases the stress over the element drops to zero which is not accurate since it is known that viscoelastic materials (e.g., polymers) retain internal stresses in the chains that are not relaxed over time [26]. The results also indicate the existence of a single relaxation time (*c*_d_/*k*) which is reflected in the inflection point in Figure 1b. The existence of a single relaxation time is also considered a limitation in depicting true viscoelastic surfaces, which generally have more than one relaxation time [27]. Finally, it is worth mentioning that although a Linear Maxwell arm might appear to be too simplistic, there may be samples whose recovery is so slow that their response could be approximately mimicked by this model [26].

#### Linear Kelvin–Voigt model

**Figure 2:**
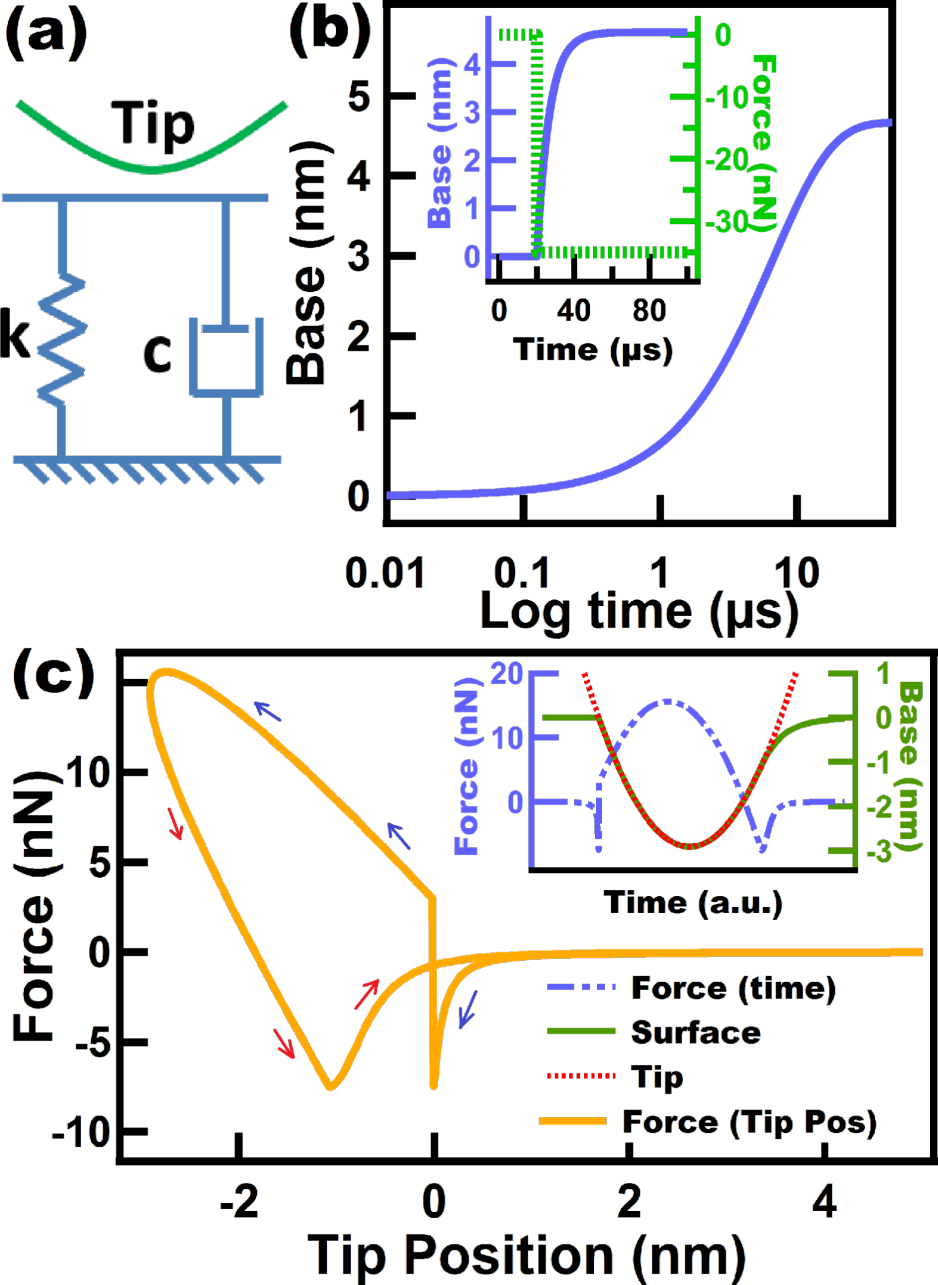
(a) Linear Kelvin–Voigt model scheme; (b) creep simulation performed on a Linear Kelvin–Voigt surface, whereby a force of −35 nN is applied at time zero. The horizontal axis is plotted using a logarithmic scale to show the inflection point corresponding to the single retardation time of the model. The inset shows the same experiment but here the force is applied at time *t* = 20 µs and the surface creeps. (c) Force–distance tip trajectory corresponding to a tapping tip over a Linear Kelvin–Voigt sample. The inset shows the base (surface) and tip position, and the force along one fundamental oscillation. The tip was oscillated along a numerically simulated trajectory (not prescribed) for tapping mode AFM. The parameters used for (c) are: cantilever position *z*_c_ = 80 nm, natural frequency (*f*_0_) = 75 kHz, free amplitude (*A*_01_) = 100 nm, cantilever stiffness (*k*_m1_) = 4 N/m. The Linear Kelvin–Voigt parameters used for (b) and (c) were *k* = 7.5 N/m and *c* = 1.0 × 10^−6^ N·s/m. The blue and red arrows in (c) correspond to approach and retraction of the tip, respectively.

Another simple model comprised by a spring and a dashpot in parallel is known as the Linear Kelvin–Voigt model (Figure 2a). This model is known for successfully describing creep compliance, but failing to describe stress relaxation. The surface lacks a spring that is able to accommodate the immediate force applied to it. Instead, the only spring in the model does not have an immediate response and it only experiences compression until the parallel dashpot starts yielding. As a result, a sudden step appears in the FD curve in Figure 2c upon impact. The magnitude of the step in the force (*F*) will depend on the instantaneous velocity (*v*) of the tip when it hits the sample and the viscous coefficient of the damper (*c*_d_), since the force in a linear dashpot is given by *F* = *c*_d_ *× v*. Since the tip approaches the sample with a velocity governed by the imaging and cantilever parameters, the sample surface experiences an instantaneous velocity *immediately* upon contact, which gives rise to the sudden jump in the FD curve. This is an obvious problem precluding the application of this model to tapping mode AFM. This artifact can also be seen in the inset of Figure 2c which shows the force as a function of time as well as the position of the surface and tip trajectory in time. It can be seen that the discontinuous increment of the force occurs at the moment when the probe encounters the surface.

Figure 2c shows the creep experiment on a Linear Kelvin–Voigt surface. In the inset of the figure, force and surface position are plotted as a function of the time. In this experiment a downward force of 35 nN is applied to the surface, after which the surface immediately starts creeping. When the sample retracts the model behaves as if an upward force is being applied to the surface, which causes the surface to creep back up to its original unperturbed position. This ability of the Linear Kelvin–Voigt model to reproduce creep provides a mechanism for the surface to recover to its original position, which is a feature that is not available in Linear Maxwell surfaces, as previously discussed. It is interesting to see in the inset of Figure 2c that during tip retract the surface does not seem to creep right away but instead it appears that the sample has an initial elastic response and only afterwards exhibits creep behavior, starting when the tip–sample contact is lost. The reason for this is that the surface actually creeps from the beginning but with a higher rate than the tip velocity, so in the simulations a restriction needs to be imposed to keep the surface from overtaking the tip position. As a result, the surface only creeps freely when there is no restriction by the tip, which occurs when the tip leaves the surface. As expected, for higher values of *c*_d_ (a less yielding dashpot) the creep phenomenon can be seen from the beginning of the tip retraction because the dashpot creep rate is lower than the tip velocity (Figure S1, Supporting Information File 1). In Figure 2b it can also be seen that the Linear Kelvin–Voigt model only provides one retardation time (the inflection point in the strain–log time curve). The inability of these simple models (Linear Maxwell and Linear Kelvin–Voigt) to capture multiple relaxation and retardation times constitutes a disadvantage when modeling the actual behavior of polymers and in particular when interpreting AFM data, whereby the cantilever and imaging parameters may be such that they favor only a particular relaxation time of the sample or none at all.

Despite the above disadvantages of the Linear Kelvin–Voigt model, it has been previously used in tapping mode AFM, both in experimental and numerical simulation approaches [16–17]. This model is also customarily used in contact-mode methods [28–29], for which there is no transition between contact and noncontact regimes as in tapping mode, so the sudden force artifact discussed above does not occur.

#### Standard Linear Solid (SLS) model

**Figure 3:**
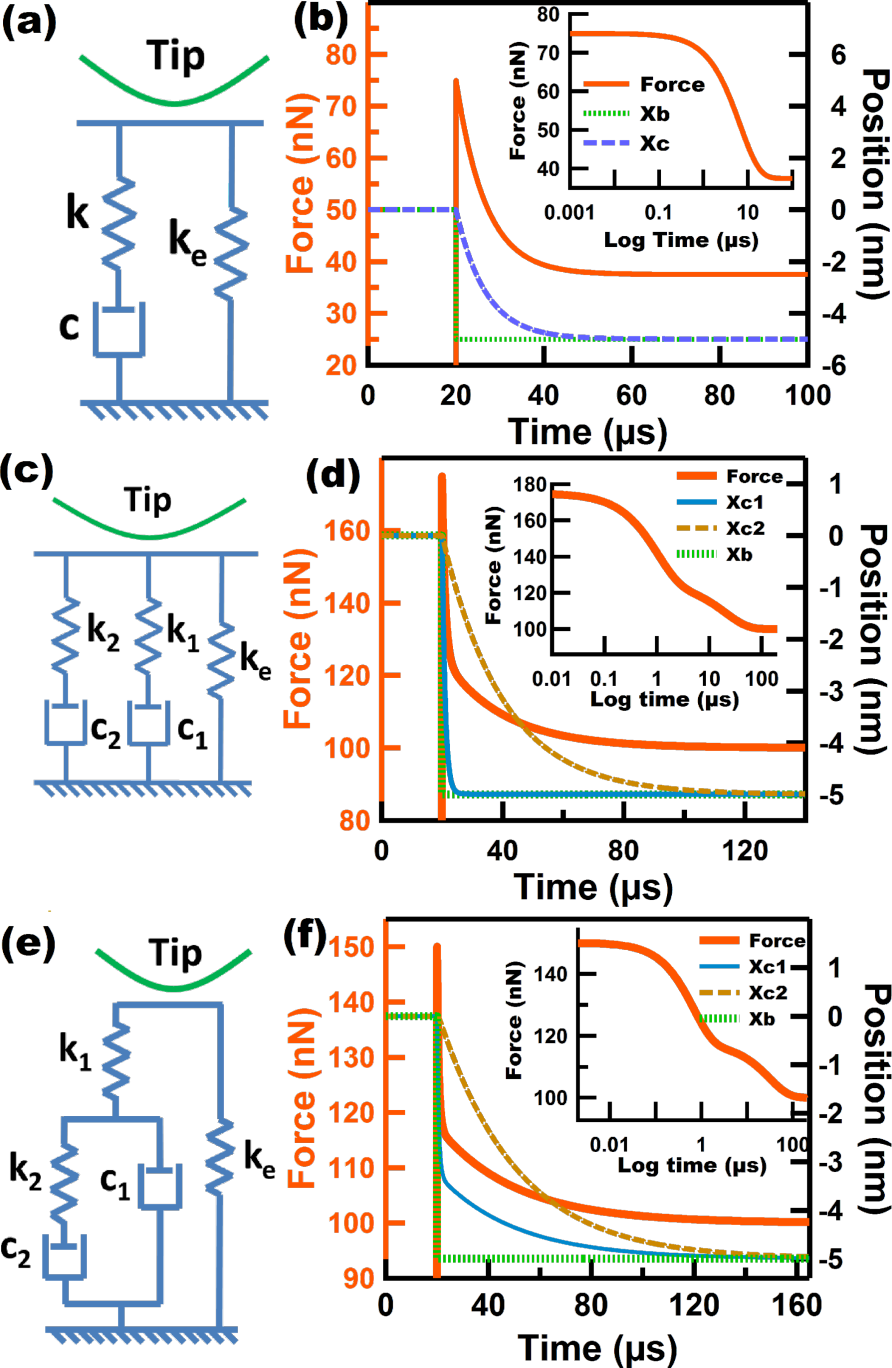
(a), (c), and (e) Standard linear solid (SLS) model, Wiechert model, and Nafion model, respectively; (b), (d), and (f) stress relaxation simulations for SLS, Wiechert, and Nafion models, respectively. The insets show the stress relaxation experiments for which time is plotted in logarithmic scale to show the inflection points corresponding to the relaxation times. The parameters for (b) are: *k*_e_ = *k* = 7.5 N/m, *c* = 5.0 × 10^−5^ N·s/m. The parameters for (d) and (f) are: *k*_e_ = 20 N/m, *k*_1_ = 10 N/m, *k*_2_ = 5 N/m, *c*_1_ = 1.0 × 10^−5^ N·s/m, *c*_2_ =10.0 × 10^−5^ N·s/m.

The SLS model is recognized as being the simplest one that is able to capture both stress relaxation and creep compliance, which are basic time-dependent properties exhibited by viscoelastic surfaces. It is comprised by a Linear Maxwell arm arranged in parallel with a spring (Figure 3a) and has been recently used in the context of multifrequency and spectral inversion AFM simulations [22–24]. Figure 3b illustrates the time-dependent properties of an SLS surface, which captures the advantages of the Linear Maxwell and Linear Kelvin–Voigt models, but exhibiting important differences in the time-dependent experiments. Figure 3b illustrates a relaxation experiment for the SLS model. Here, a restoring force of 75 nN is immediately obtained when the surface is displaced by 5 nm at time 20 µs. Then, the system relaxes through the dashpot located in the Linear Maxwell arm. However in the case of SLS the stress does not relax to zero, but rather, some stress remains stored in the spring parallel to the Linear Maxwell arm (*k*_inf_) which in this 1-dimensional case corresponds to a force of 37.5 nN. This behavior is more physically accurate for samples such as polymers, for which it is known that a total relaxation of the stress does not occur [26]. On the other hand, for the creep simulation the SLS shows an immediate response of the surface (attributed to the elastic part) right when the force is applied, before noticeable surface creep occurs (Figure S2, Supporting Information File 1). The above is not observed in the creep simulation of the Linear Kelvin–Voigt surface where creep occurs without showing an immediate elastic response (Figure 2b). In the context of tapping mode AFM the SLS also has advantages when compared to the previous models discussed. First, it provides a mechanism to accommodate the initial force through its springs during tip approach without causing the discontinuous increase in force exhibited by the Linear Kelvin–Voigt model. Second, it provides a mechanism for surface recovery which allows the surface to return to its unperturbed position (this feature is not available in the Linear Maxwell model). Despite the advantages of the SLS model, however, it does not reproduce multiple relaxation times nor nonlinear elastic behavior.

#### Wiechert model

Modeling of multiple relaxation times has generally been carried out by representing a viscoelastic surface as a series of Linear Maxwell arms in parallel with an equilibrium spring that keeps a residual stress that does not relax in time. This generalized model is known as the Wiechert model. Multiple relaxation times in a real sample are attributed to the presence of molecular segments with different lengths which have different contributions [30]. Figure 3c shows a Wiechert model with two Linear Maxwell arms. We have chosen this particular configuration for simplicity, as the main goal is to illustrate its application in the context of tapping mode AFM. Figure 3d shows a stress relaxation simulation for the Wiechert model chosen, and as expected, the existence of two relaxation times is evidenced by the presence of two inflection points in the inset. Each relaxation time is linked to the relaxation of each of the Linear Maxwell arms. The dashpot constants were intentionally chosen to have significantly different values (*c*_1_ = 1.0 × 10^−5^ N·s/m and *c*_2_ = 10.0 × 10^−5^ N·s/m) in order to more clearly show the presence of the multiple relaxation times. In the context of tapping mode AFM this model exhibits a behavior that is qualitatively similar to that of the SLS model. That is, it is able to successfully accommodate the initial force experienced by the surface during the approach of the tip and also provides a mechanism for surface recovery through the stress stored in the equilibrium spring. The FD curve of the Wiechert model (Figure S3, Supporting Information File 1) also looks qualitatively similar to the FD curve of the SLS model. Both are characterized by the presence of two minima and a dissipation loop, and both curves are smooth with no discontinuity artifacts as for the Linear Kelvin–Voigt model.

#### Nafion^®^ model

The Nafion model was introduced by Boyce and coworkers [31] to mimic the behavior of the Nafion proton exchange polymer in biaxial loading tests. This model, shown in Figure 3e, consists of a standard linear fluid element (a Linear Maxwell arm in parallel with a *dashpot*) in series with a spring and in parallel with an equilibrium spring. The special arrangement in this model attempts to reproduce the molecular and intermolecular rearrangement that Nafion undergoes during the application of stress [31], which motivated us to consider it in the context of AFM. However, it is important to point out that the original model has nonlinear springs and dashpots, whereas the model illustrated here only contains linear elements. This has been done for simplicity, but one must be mindful that nonlinear elements should be present to account for the geometrical aspects of the changing tip–sample contact area during impact. A stress relaxation simulation of the Nafion model is shown in Figure 3f. The inset clearly indicates the presence of two relaxation times in the force–log time curve. Is interesting to see that the rate at which the force drops is proportional to the rate of motion of dashpot *c*_1_. The above is explained by the fact that the drop in the force in spring *k*_1_ is dictated by the motion of dashpot *c*_1_ and, at the same time, the relaxation of the entire model is governed by spring *k*_1_, since the equilibrium spring never relaxes. Is also interesting to mention that the Linear Maxwell arm initially experiences an increase in force, after which the force starts to drop. In this model, as in the two previously described models, the force does not fall to zero but instead reaches a minimum force stored in the equilibrium spring *k*_e_. This model exhibits a very interesting behavior under the influence of a tapping tip, which is discussed in the second part of this study.

#### Nonlinear models

Typically, viscoelasticity in the context of AFM has been modeled by the addition of a dissipative force term (*F*_ts_^DISS^*)* to the conservative force term(s) (*F*_ts_^CON^), such that the total tip–sample force can be expressed as *F*_ts_ = *F*_ts_^CON^ + *F*_ts_^DISS^. Usually the repulsive conservative portion of the tip–sample interaction force is defined through the Derjaguin–Muller–Toporov (DMT) model or a similar model [6,32] while the conservative attractive force corresponding to van der Waals interactions is modeled through the Hamaker equation [6]. The dissipative portion of the interaction has been typically modeled through a velocity-dependent term. One approach (often used by us) has been to use the model of Gotsmann and coworkers [33], in which the dissipative force, *F*_diss_, is proportional to the negative of the tip velocity (that is, acting in opposite direction) through an exponentially decaying coefficient:

[1]
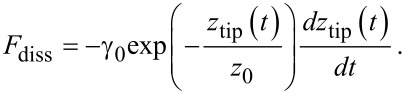


**Figure 4:**
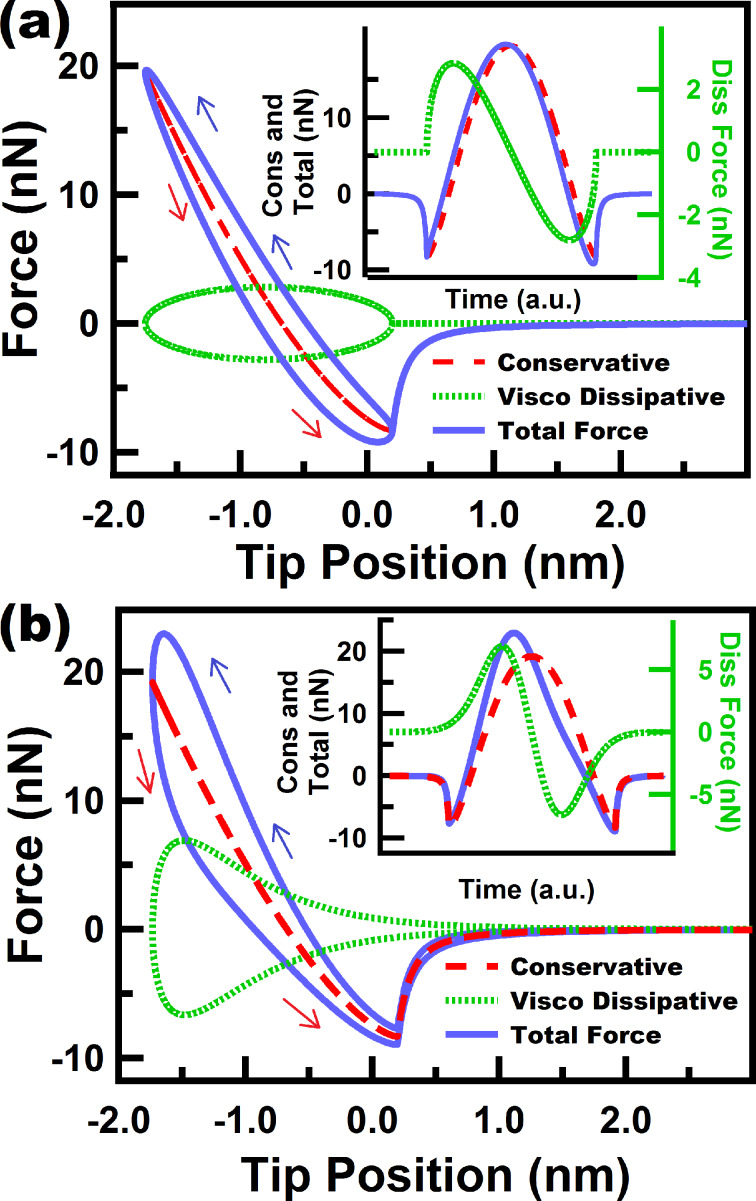
(a) and (b) show force trajectories for a tip under a numerically simulated (not prescribed) single tapping mode trajectory for a DMT-García sample and a DMT-Gotsmann sample, respectively. The figures show the individual contribution of the conservative and dissipative forces, along with the total force. The insets show the behavior of the force contributions over time and reflect the symmetry aspects discussed in the main text. The simulation parameters for the cantilever dynamics in (a) and (b) are: cantilever position *z*_c_ = 80 nm, natural frequency (*f*_0_) = 50 kHz, free amplitude (*A*_01_) = 50 nm, cantilever stiffness (*k*_m1_) = 10 N/m. The model parameters for (b) and (c) associated with the DMT contribution are: elastic sample modulus (*E*_s_) of 3 GPa, elastic tip modulus (*E*_t_) of 150 GPa, Poisson’s ratio of tip and sample (υ_t_ and υ_s_, respectively) of 0.3, tip radius of curvature (*R*) of 10 nm, Hamaker constant of 2 × 10^−19^ J. For (a) a viscosity (η) value of 400 N·s/m^2^ was used. For (b) a dissipation coefficient (γ_0_) of 3 × 10^−7^ kg/s, and a characteristic dissipation length (*z*_0_) of 0.75 nm were used. The blue and red arrows correspond to approach and retraction of the tip, respectively.

Here *z*_tip_ is the instantaneous cantilever tip position, γ_0_ is a dissipation coefficient with units of mass*/*time and *z*_0_ is a characteristic length over which the dissipation force decays (in this study we will refer to this model as ‘DMT-Gotsmann’). Another very widely used approach to incorporate the dissipative portion of the tip–sample forces, is an adapted Voigt model that uses Hertz contact mechanics to incorporate the contact area and sample deformation [7]. The dissipative part of this model, originally introduced by García and coworkers [34] has the following form:

[2]
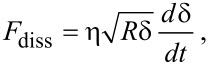


where η is the viscosity, *R* is the tip radius and δ is the sample deformation (tip–sample indentation). In this model, the linear spring in the Linear Kelvin–Voigt material is also replaced by a nonlinear DMT spring. As a result, this model is able to capture the nonlinear behavior of the tip–sample interactions (this model will be referred to in this study as DMT-García). Typical FD curves of the above two models are shown in Figure 4, in which we show the conservative and dissipative contributions along with the total force. The insets illustrate how the different contributions of the force behave in time. It can be clearly seen that the conservative part is symmetric, the dissipative part is antisymmetric and the total force lacks symmetry. Both approaches (DMT-Gotsmann model and DMT-García) share a similar concept in which the viscous force is proportional to the deformation velocity, and also both include varying contributions due to contact area variations. One important difference is that the DMT-Gotsmann model includes viscoelastic contributions in the conservative *attractive* part of the interaction, which is generally not physically meaningful for viscoelastic surfaces since viscoelastic dissipation only occurs in the contact regime, although there can be other contributions to dissipation in the non-contact regime such as long-range and short-range surface adhesion [7], the discussion of which is beyond the scope of this study. One important disadvantage of the DMT-Gotsmann and DMT-García models is that neither is able to reproduce the fundamental rate-dependent properties of viscoelastic materials, namely stress relaxation and creep. Furthermore, these models do not mimic the behavior of the sample but instead always assume a static unperturbed sample. As a result the FD curves only show one minimum at the fixed point where the tapping probe always finds the sample. On the other hand, one significant advantage of these models over the linear models discussed in previous sections is that they take into account the effect of a varying contact area on the stiffness and dissipative coefficient of the tip–sample interaction.

**Figure 5:**
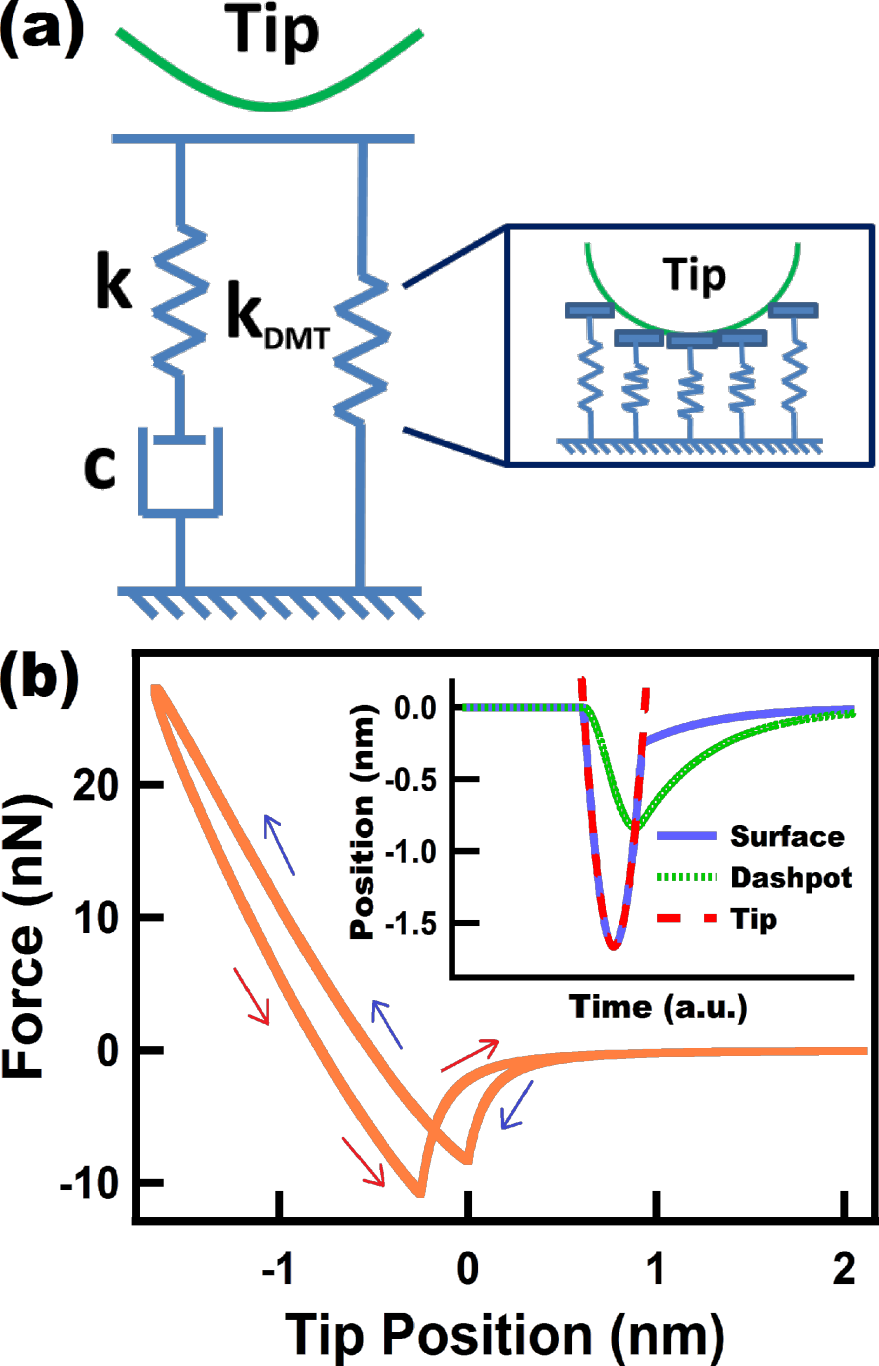
(a) Standard nonlinear solid (SNLS) model. (b) Tip force trajectory for a tip under a numerically simulated (not prescribed) tapping mode trajectory interacting with a SNLS sample. The inset in (b) shows the position of the surface and the dashpot over one fundamental oscillation. The parameters for the cantilever dynamics were the same as in Figure 2b. The parameters for *k*_DMT_ were the same as in Figure 4. The parameters *k* and *c* were set to 7.5 N/m and 0.5 × 10^−5^ N·s/m, respectively. The blue and red arrows in (b) correspond to approach and retraction of the tip, respectively.

As an initial attempt to blend the advantages of the linear spring–dashpot models with the advantages of the current nonlinear models used in AFM, we propose here a standard nonlinear solid (SNLS). Figure 5a shows a diagram of the SNLS. The structure resembles the SLS but it incorporates a nonlinear DMT spring as the equilibrium spring. This configuration was chosen because the SLS is the simplest model that is able to describe stress relaxation and creep, and the DMT is a widely used model in contact mechanics that is typically used in the context of AFM. We include both DMT contact forces and long-range van der Waals forces [6,32].

[3]
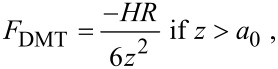


[4]
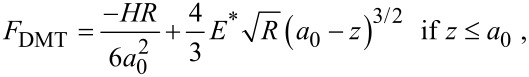


[5]
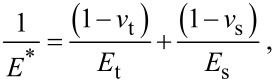


where *H* is the Hammaker constant, *R* is the tip radius, *z* the tip position with respect to the sample, *a*_0_ the intermolecular distance, E* the effective tip–sample elastic modulus, *E*_t_ and *E*_s_ the elastic modulus of tip and sample, respectively, and ν_t_ and ν_s_ are the Poisson’s ratios of the tip and the sample, respectively.

In general, the SNLS works similar to the SLS with the difference that the SNLS includes a DMT element as the equilibrium spring. In Figure 5a we provide a physical representation of the SNLS model in which the DMT spring can be visualized as an infinite collection of springs, for which the number of active elements increases as the tip goes deeper into the sample. The above is mathematically represented with a nonlinear spring whose stiffness depends on the position of the tip and the contact area [32]. A typical FD curve for this model is shown in Figure 5b, in which the nonlinear behavior of the contact region is evident, along with the presence of two force minima, as in the spring–dashpot models.

#### Dissipation-based analysis

Extracting material properties in a fast and accurate way is one of the ultimate goals in AFM. In order to accomplish this for viscoelastic surfaces, physically accurate models are a requirement. One very distinct feature of viscoelastic materials is that they dissipate energy when they are subjected to dynamic loading. In the particular case of tapping mode AFM, the sample experiences a cyclic, nearly sinusoidal loading. For tapping mode AFM, other authors have derived expressions that link the observables (phase and amplitude) to the energy dissipated [9,35], and these relationships have been widely used [10–11,36–37]. Although the amount of dissipation is an important hint to the nature of the material, it is not possible to derive unambiguous conclusions about the material properties by probing a material with a single condition. With viscoelasticity being a rate-dependent phenomenon, it becomes necessary to characterize the sample at different velocities, which is not trivial in practice for tapping mode AFM. Such characterization would require imaging the sample with cantilevers of different fundamental frequencies or the use of different higher eigenmodes in successive experiments [22]. For this numerical study, we have chosen the first approach and for simplicity we have restricted ourselves to single-eigenmode tapping mode AFM due to the introductory nature of this work and to keep it from becoming excessively lengthy.

**Figure 6:**
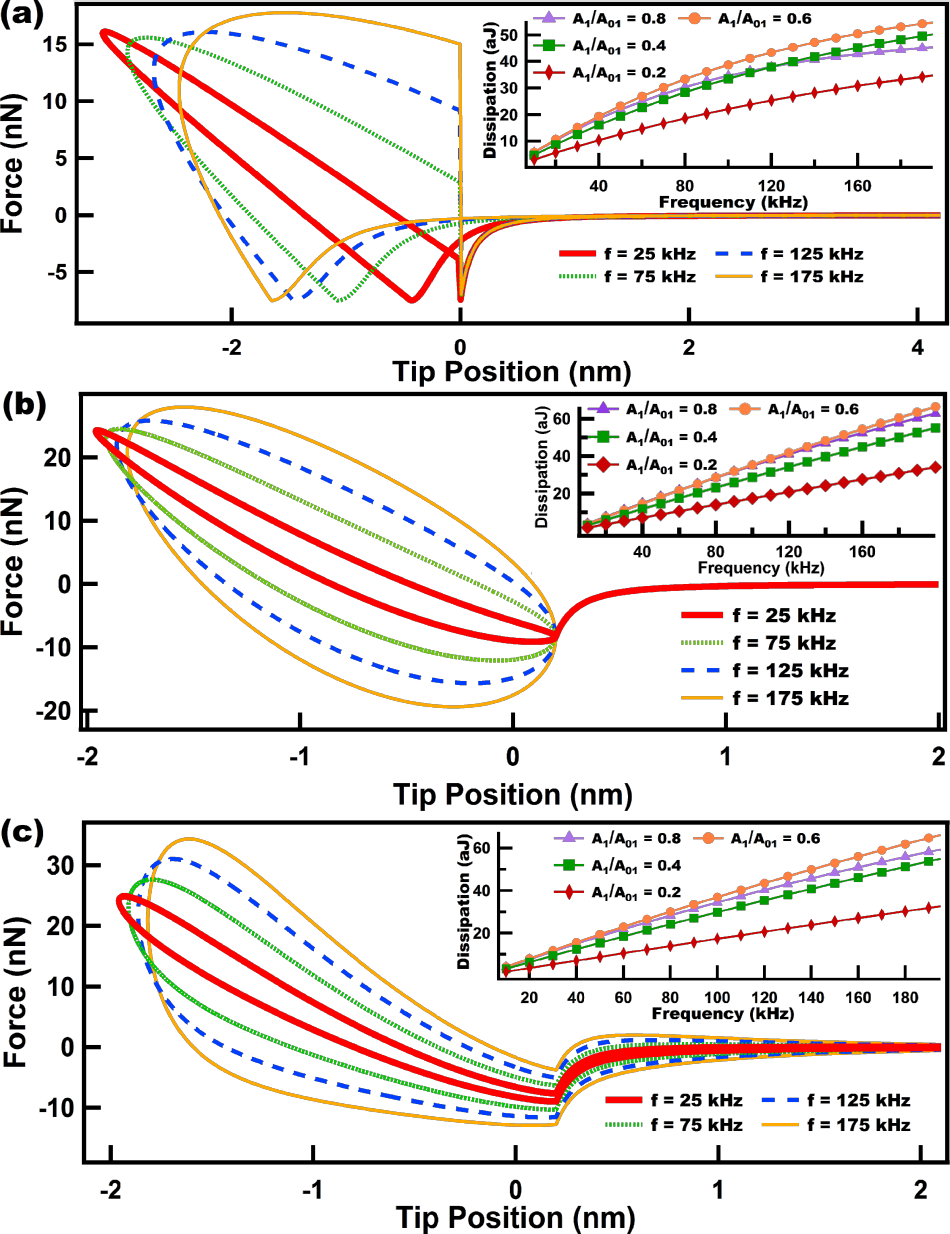
(a), (b) and (c) show force trajectories for a tip following a numerically simulated (not prescribed) single tapping mode trajectory over a Linear Kelvin–Voigt, a DMT-García, and a DMT-Gotsmann sample, respectively. Each of the force trajectories is color-coded according to the cantilever eigenfrequency used in the simulation. The insets in the figures show the behavior of dissipation as the frequency increases, and each color coded line relates to a specific amplitude setpoint (*A*_1_/*A*_01_). The simulation parameters for the cantilever dynamics in (a), (b) and (c) are: cantilever position *z*_c_ = 80 nm, free amplitude (*A*_01_) = 100 nm, cantilever stiffness (*k*_m1_) = 4 N/m. The Linear Kelvin–Voigt parameters for (a) were *k* = 7.5 N/m and *c* = 1.0 × 10^−6^ N·s/m. The sample parameters for (b) and (c) are the same as for Figure 4.

Dissipation in AFM has often been studied by using dissipation vs amplitude setpoint (*A*_1_/*A*_01_) curves, in which it has been possible to distinguish differences in dissipation arising from viscoelasticity, long- and short-range interactions [34]. We have also followed this approach in the current study. Figure 6 shows the FD behavior of three different models (Linear Kelvin–Voigt, DMT-García and DMT-Gotsmann) for different cantilever frequencies, with the insets showing the corresponding dissipation behavior for different frequencies and different amplitude setpoints (ratio of engaged amplitude to free amplitude *A*_1_/*A*_01_). We have analyzed these models together since they share some common features. For the Linear Kelvin–Voigt model (Figure 6a) the dissipation has a direct relation with the velocity, through the linear viscous dashpot in its structure (Figure 2a). For this simple model, which does not include stress relaxation, dissipation arises from the presence of a viscous linear dashpot where the magnitude of the dissipation force is related to the velocity by the simple relation 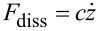. For the other two models in Figure 6, dissipation is incorporated through a nonlinear dashpot, but still the emergence of dissipation is in the form of 
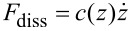
. All the results in Figure 6 show that, regardless of the setpoint, dissipation grows monotonically over the entire range of frequencies studied.

The models in Figure 6 share some common features and the nonlinear models (Figure 6b and Figure 6c) can be considered enhancements of the simple linear Linear Kelvin–Voigt model, which are able to capture the nonlinear interactions of the probe in intermittent contact with the sample. However, we again remind the reader that these models do not exhibit stress relaxation, and in the case of DMT-Gotsmann and DMT-García, they do not exhibit creep either. Nonetheless, the DMT-García model has been able to successfully describe certain viscoelastic materials under specific conditions [21], and even the simple Linear Kelvin–Voigt model has shown applicability in tapping mode AFM for the calculation of loss tangent in simulations [16], although it has shown to be inaccurate in experimental applications, in which real samples are involved [17].

**Figure 7:**
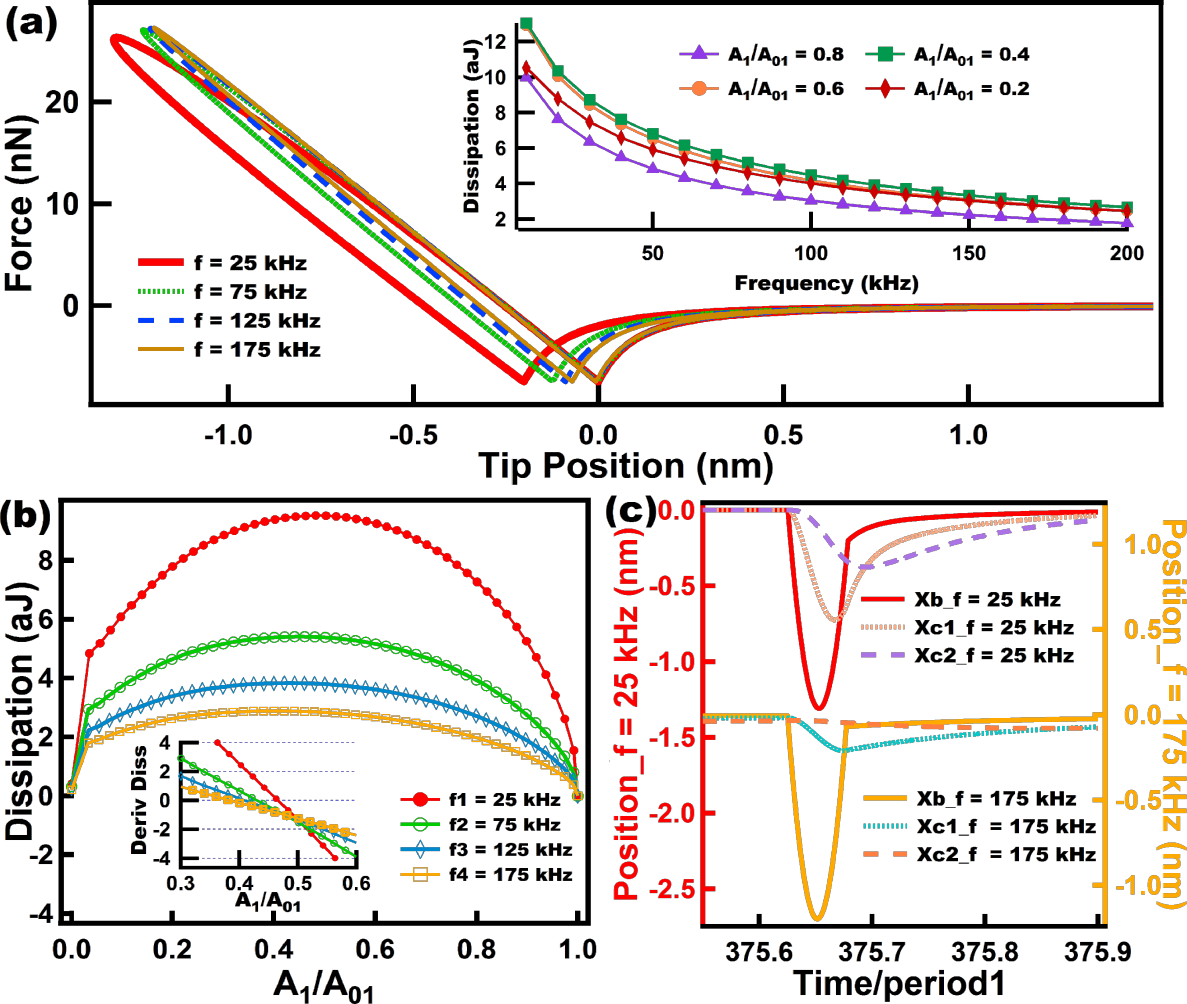
Results of energy dissipation when a numerically simulated tip trajectory in intermittent contact AFM interacts with a Nafion model. (a) shows force–distance curves for tips driven at different eigenfrequencies (color coded). The inset in (a) shows the behavior of the dissipation as the frequency grows, and each color-coded line relates to a specific amplitude setpoint (*A*_1_/*A*_01_). (b) shows dissipation vs amplitude setpoint (*A*_1_/*A*_01_) curves in which each color-coded line corresponds to a specific eigenfrequency. The inset in (b) shows the slope of the curves in (b) near the range where the slopes cross the *x*-axis in order to easily locate the maxima of the curves in (b). (c) shows the response of the surface and dashpots of the Nafion model for two cases at different cantilever eigenfrequencies. The parameters for the cantilever dynamics are the same as in Figure 6. The sample parameters use were: *k*_e_ = 20 N/m, *k*_1_ = 10 N/m, *k*_2_ = 5 N/m, and *c*_1_ = *c*_2_ = 1.0 × 10^−5^ N·s/m. Time normalization has been carried out in (c) with respect to the fundamental period for ease of comparison.

**Figure 8:**
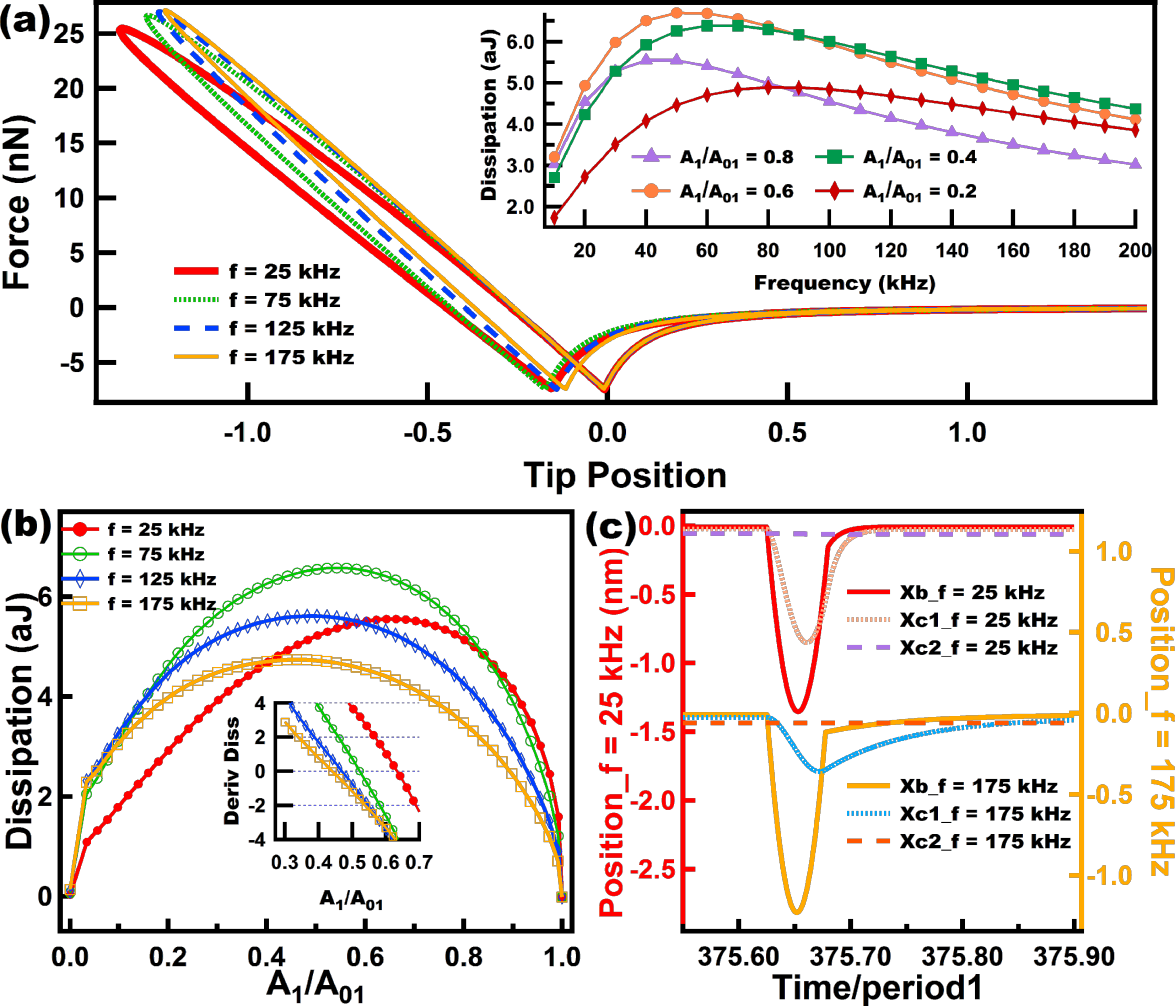
Results of energy dissipation when a tip interacts with a Nafion model under a numerically simulated trajectory. (a) Shows force distance curves for tips driven at different eigenfrequencies (color coded). The inset in (a) shows the behavior of dissipation as the frequency increases, and each color coded line relates to a specific amplitude setpoint (*A*_1_/*A*_01_). (b) shows dissipation vs amplitude setpoint (*A*_1_/*A*_01_) curves, where each color coded line corresponds to a specific eigenfrequency. The inset in (b) shows the slope of the curves in (b) near the range where the slopes cross the *x*-axis in order to easily locate the maxima of the curves in (b). (c) shows the response of the surface and dashpots of the Nafion model for two cases at different cantilever eigenfrequencies. The parameters for the cantilever dynamics were the same as in Figure 6 and Figure 7. The sample parameters were: *k*_e_ = 20 N/m, *k*_1_ = 10 N/m, *k*_2_ = 5 N/m, *c*_1_ = 0.5 × 10^−5^ N·s/m, and *c*_2_ = 100.0 × 10^−5^ N·s/m. Time normalization has been carried out in (c) with respect to the fundamental period for ease of comparison.

In Figure 7 and Figure 8 we have varied the parameters of the Nafion model in order to tune the importance of the dashpot elements in the viscoelastic model. In this type of model the dissipation per cycle is related to the magnitude of the dashpot constant, where larger dashpot constants lead to lower dissipation (recall that here there are springs that accommodate the immediate response of the cantilever). This does not apply to the case of the Linear Kelvin–Voigt model because there are no springs to accommodate the immediate sample deformation induced by the tip. In the case of the Linear Kelvin–Voigt and other models, in which the dashpot immediately experiences the sample deformation (e.g., in the standard linear fluid element (dashpot in parallel with a Linear Maxwell arm)), the amount of dissipation is proportional to the constant (dissipation coefficient) of the dashpot that immediately experiences strain upon deformation. This discrepancy comes from the fact that the mechanism for the emergence of dissipation for models that accommodate initial response through springs (e.g., standard linear solid and Linear Maxwell) and those that do not (e.g., Linear Kelvin–Voigt and standard linear fluid) is fundamentally different. The latter ones experience immediate viscous dissipation whenever there is a surface displacement while the former experience dissipation through subsequent surface relaxation of stress initially stored in springs.

In the case of the Nafion model we have varied the magnitude of *c*_1_ and *c*_2_ (see Figure 3a) to observe the effect of changing the relative importance of the damping elements. Figure 7 shows the results for the case when both dashpots have the same damping constant. Figure 7a illustrates how dissipation decreases when the frequency increases for the range studied here (10–200 kHz). It is interesting to see in Figure 7b that regardless of the amplitude setpoint (*A*_1_/*A*_01_) the level of dissipation was higher for lower frequencies along the entire range of parameters studied here. That is, there is no overlap of the dissipation vs amplitude setpoint curves for different frequencies in Figure 7b. The slope of the dissipation vs amplitude setpoint curves is an important parameter in characterizing dissipation [34], which also facilitates the determination of the maximum in these curves. The inset in Figure 7b was plotted for a range in which it is easy to inspect where the derivative crosses the *x*-axis. For this case the maximum of the curves slightly shift to the left as the frequency increases within a range of 0.4 to 0.5 of the ratio *A*_1_/*A*_01_.

In contrast, for Figure 8, when the dashpot *c*_2_ is set to a high damping value compared to *c*_1_ (notice that the dashpot *c*_2_ in Figure 3a hardly yields when compared to *c*_1_) the behavior of dissipation changes drastically compared to the results of Figure 7. In the inset of Figure 8a it can be seen that for the range of frequency studied, for every setpoint dissipation increases until it reaches a certain maximum, depending on the setpoint, and then decreases to lower values. In dynamic loading experiments in the polymer literature this maximum is related to a glass transition temperature *T*_g_ where the loss modulus (which is proportional to dissipation) peaks within that frequency range [27]. Figure 8b confirms the trend in the inset of Figure 8a. It can be seen that from 25 kHz to 75 kHz dissipation increases almost for all the setpoints (except for high setpoints) and then decreases for higher frequencies (from 75 kHz to 175k Hz) almost for all setpoints (except for low setpoints where *A*_1_/*A*_01_ is lower than 0.15). The inset in Figure 8b shows that the maximum of the dissipation vs setpoint curve shifts to the left as the frequency increases. The behavior of this model (Figure 8) is more intricate than the one shown in Figure 7 and illustrates the challenges in choosing the ideal parameters when an experimentalist wants to maximize dissipation in tailoring a material that follows this model.

**Figure 9:**
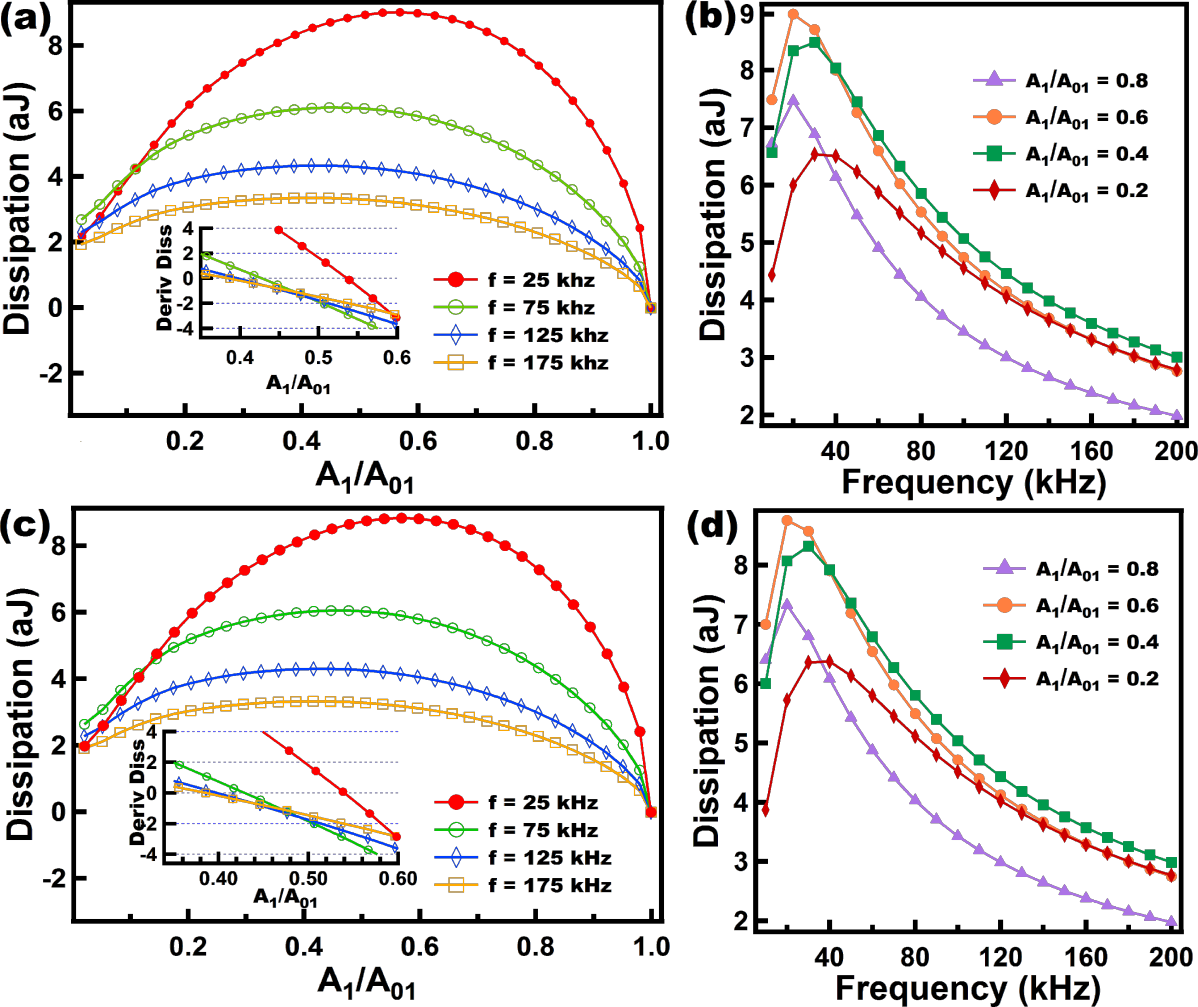
(a) and (c) show dissipation vs amplitude setpoint (*A*_1_/*A*_01_) curves where each color coded line corresponds to a specific eigenfrequency, for the Wiechert and the SLS models, respectively. The insets in (a) and (c) show the slope of the curves in (a) and (c) plotted over a range near where the slopes cross the *x*-axis, in order to easily locate the maxima of the curves in (a) and (c). (b) and (d) show the behavior of dissipation as frequency increases, and each color coded line relates to a specific amplitude setpoint (*A*_1_/*A*_01_) for the Wiechert and the SLS models, respectively. The parameters for the cantilever dynamics were the same as in Figures 6 to 8. The sample parameters for (a) and (b) were: *k*_e_ = 10 N/m, *k*_1_ = 7.5 N/m, *k*_2_ = 7.5 N/m, *c*_1_ = 0.5 × 10^−5^ N·s/m, and *c*_2_ = 100.0 × 10^−5^ N·s/m. The sample parameters for (c) and (d) were: *k*_e_ = 17.5 N/m, *k* = 7.5 N/m, *c* = 0.5 × 10^−5^ N·s/m.

Figure 9 shows dissipation behaviors when performing tapping mode AFM over two different viscoelastic samples: Figure 9a and Figure 9b show results for the Wiechert model, and Figure 9c and Figure 9d for the SLS model. For this set of simulations the Wiechert model (see diagram in Figure 3c) had a value of *c*_2_ that was much higher than the value of *c*_1_, with the purpose of making *c*_2_ less relevant in terms of the amount of dissipation observed. Afterwards, an SLS model was simulated with parameters that approximate the response of the more complex Wiechert model (see the caption of Figure 9). The dissipation results show very similar trends for both models and also a close quantitative agreement between both, which is reasonable since the Wiechert model was set in a way that one of the dashpots dominates during the intermittent tip–sample interactions, and as previously stated, the mechanism of dissipation of this spring–dashpot models is surface relaxation which happens when the force, built up in the springs, drops through the yielding of the dashpots. There is still a small difference between the models, which can be observed in Figure 9b and Figure 9d where the values of the dissipation are slightly higher for the Wiechert model. This difference is attributed to the modest contribution of dissipation arising from the stress relaxation that occurs through *c*_2_ in the Wiechert model.

**Figure 10:**
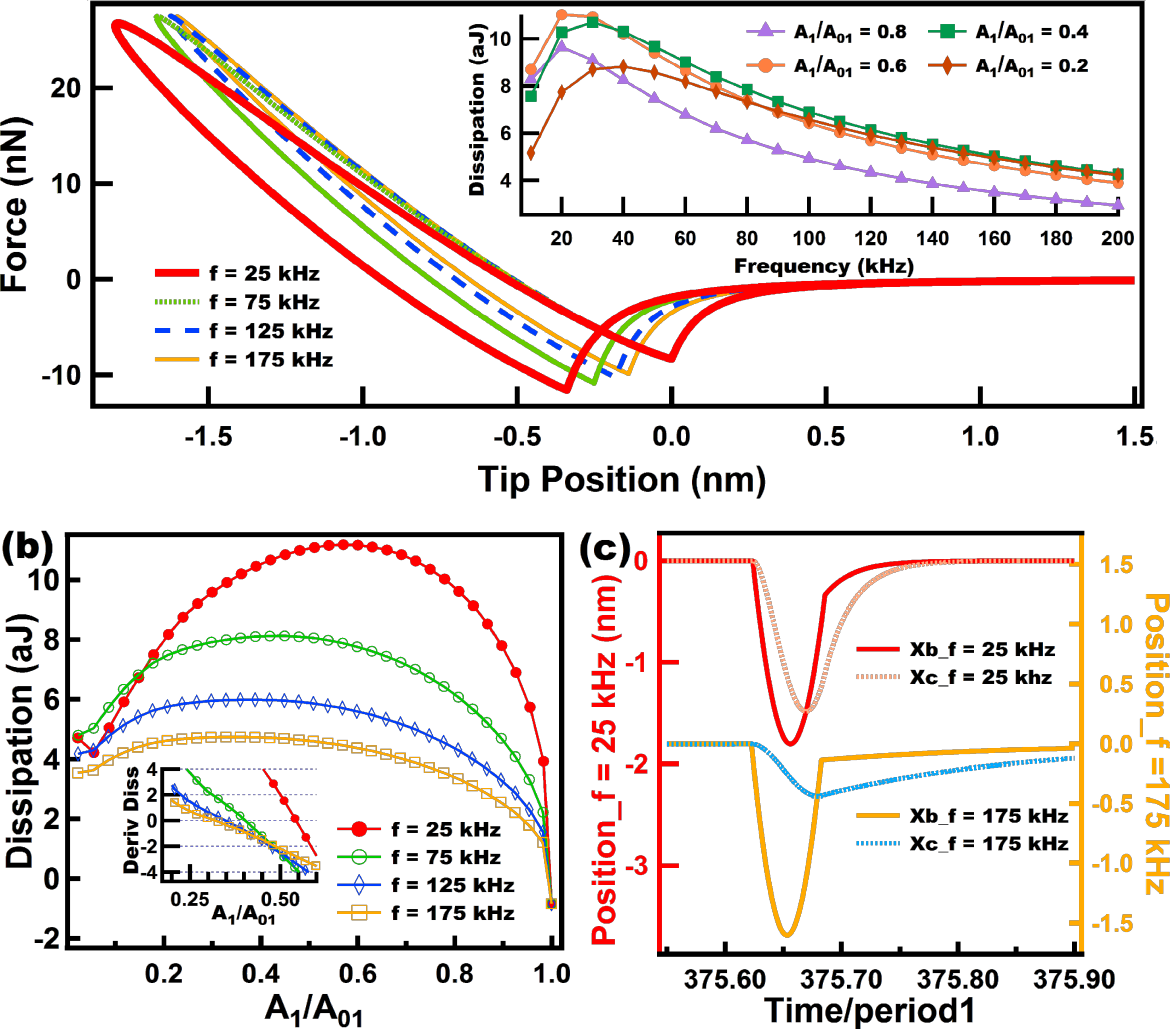
Results of energy dissipation when a realistic tip interacts in intermittent contact AFM with a standard nonlinear solid (SNLS). (a) Shows force–distance curves for tips driven at different eigenfrequencies (color coded). The inset in (a) shows the behavior of dissipation as frequency increases, and each color coded line relates to a specific amplitude setpoint (*A*_1_/*A*_01_). (b) Shows dissipation vs amplitude setpoint (*A*_1_/*A*_01_) curves where each color coded line corresponds to a specific eigenfrequency. The inset in (b) shows the slope of the curves in (b) plotted in the range where the slopes cross the *x*-axis in order to easily determine the maxima of the curves in (b). (c) Shows the response of the surface and dashpots of the SNLS model for two different cantilever eigenfrequencies. The parameters for the cantilever dynamics were the same as in Figures 6 to 9. The sample parameters were the same as for Figure 5.

Figure 10 shows dissipation results for the standard nonlinear solid model (SNLS). Figure 10a shows FD curves at different frequencies, from which it is clear that at low frequencies the dissipation loop is larger and also the tip position reaches lower values corresponding to a greater tip–sample indentation. As the tip reaches lower values, the minimum of the FD curve when the tip *leaves* the sample (the left-most minimum in the FD curves, corresponding to the tip retract) is also lower. The above is explained by the fact that lower frequencies allow for a longer time for relaxation of the dashpots. The inset in Figure 10 shows the behavior of dissipation at different frequencies, exhibiting a similar trend as for the SLS model where the two extremes of frequencies show low dissipation, coinciding with the behavior of amorphous polymers, which in dynamic loading tests are expected to undergo highly elastic behavior in the two extremes of frequencies [27]. Figure 10b also shows similar qualitative behavior to that of the SLS model, with the maxima in the inset shifting to the left for larger frequencies. Finally, Figure 10c shows the response of the surface and the dashpot of the SNLS model to different frequencies. This figure shows clearly than at 25 kHz the dashpot in the model is able to respond significantly while at 175 kHz the dashpot response is much smaller. As a result, the surface is able to experience greater surface relaxation (leading to greater dissipation) at 25 kHz.

## Conclusion

Different approaches to model viscoelasticity within intermittent contact AFM have been studied with special emphasis on spring–dashpot models. We summarize the models that have been frequently used in AFM, highlighting their strengths and deficiencies. We also propose different spring–dashpot models that can be used to mimic the response of viscoelastic surfaces, especially polymers, under interactions with the AFM tip. Most of the models included display distinct features observed in polymers, namely stress relaxation and creep, and some of them exhibit multiple relaxation times, as in realistic samples. The level of complexity and physical accuracy is different for each model and good judgment is advised in selecting the proper model for the type of sample or dynamic phenomenon under investigation. Although this paper is not intended to serve as an exhaustive manual for modeling viscoelasticity in AFM, it is our aim that it sparks further theoretical developments, which are much needed especially as new rapid AFM-based spectroscopy techniques are developed [21].

### Methods

Numerical simulations of the cantilever dynamics were performed for most of the cases according to single-eigenmode tapping mode AFM, unless otherwise indicated. To model the dynamics of the cantilever we included the contribution of the first three flexural modes of the cantilever (although only the first one was excited). Each mode was described through an individual equation of motion, and the three single equations were coupled through the tip–sample interaction forces as in previous studies [19,22,24,37–38]. The first eigenmode (the only one actively driven) was excited at its natural frequency. The first three quality factors of the cantilever were set to *Q*_1_ = 220, *Q*_2_ = 450, and *Q*_3_ = 750 in all cases, and the rest of the parameters are indicated in the figure captions for each case. The equations of motion were integrated numerically and the amplitude and phase for the first eigenmode were calculated using the in-phase (*I_i_*) and quadrature (*K_i_*) terms:

[6]
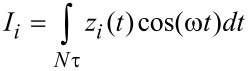


[7]
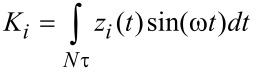


where *z_i_*(*t*) is the *i*-th eigenmode response in the time domain, *N* is the number of periods over which the phase and amplitude were averaged, ω is the excitation frequency, and τ is the fundamental period of one oscillation. The amplitude and phase (used in the dissipation analysis) were calculated, respectively, as:

[8]
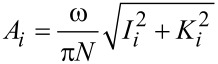


[9]
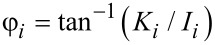


The repulsive tip–sample forces were simulated through the various models discussed throughout the paper, and the parameters for each of the models are provided in the respective figure captions.

## Supporting Information

Supporting Information features additional simulation data, namely the surface response of Linear Kelvin–Voigt samples and its dependency on its dissipation coefficient, the creep simulation of a SLS sample, and the comparison between the force–distance curves of the SLS and the Nafion model.

File 1 Additional simulation data.
