# Supplementary material for: Modeling viscoelasticity through spring–dashpot models in intermittent-contact atomic force microscopy
Source: Beilstein J Nanotechnol. 2014 Nov 18;5:2149–63. doi: 10.3762/bjnano.5.224 (PMC4273292; doi:10.3762/bjnano.5.224)
Supplement: File 1 — Additional simulation data. [file Beilstein_J_Nanotechnol-05-2149-s001.pdf]

Supporting Information

for

Modeling viscoelasticity through spring–dashpot models in intermittent-contact atomic force microscopy

Enrique A. López-Guerra and Santiago D. Solares*

Address: Department of Mechanical Engineering, University of Maryland, College Park, Maryland 20742, United States; Current Address: Department of Mechanical and Aerospace Engineering, George Washington University, Washington, DC 20052, United States

Email: Santiago D. Solares* - ssolares@gwu.edu

* Corresponding author

Additional simulation data

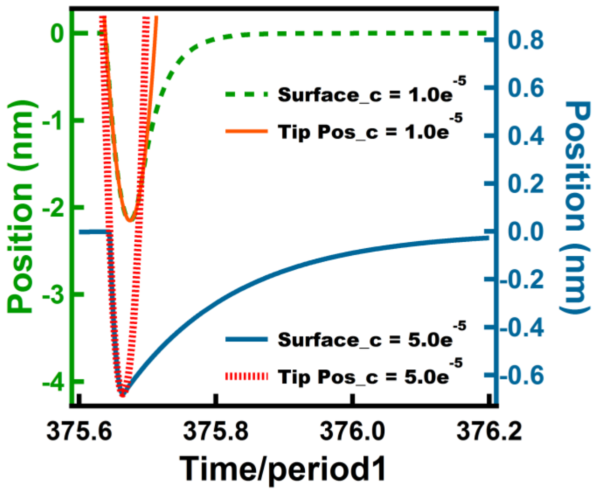

Figure S1: Surface response of two Linear Kelvin–Voigt samples interacting with the AFM tip, under a numerically integrated (not prescribed) single-eigenmode tip trajectory. The parameters used for the cantilever dynamics are: cantilever position z_c = 80 nm, natural frequency (f_0) = 25 kHz, free amplitude (A_{01}) = 100 nm, cantilever stiffness (k_{m1}) = 4 N/m. The Kelvin–Voigt parameters for the upper traces are: k = 7.5 N/m and $c = 1.0 \times 10^{-5}$ N·s/m. The Kelvin–Voigt parameters for the lower traces are: k = 7.5 N/m and $c = 5.0 \times 10^{-5}$ N·s/m.

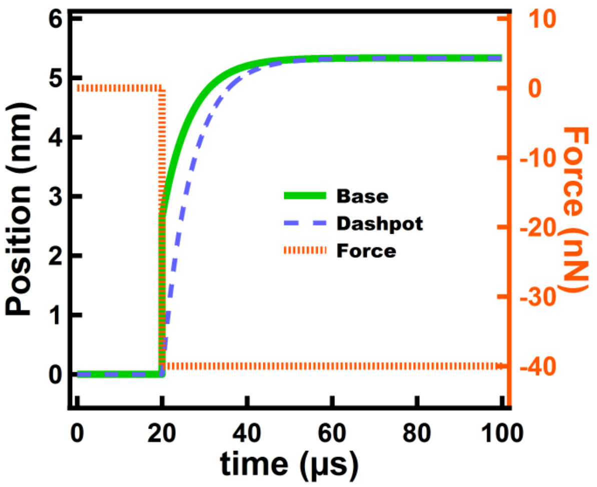

Figure S2: Creep simulation performed for an SLS surface. A downward force of 40 nN is applied at time t = 20 μs. For this model the surface experiences an immediate elastic response at t = 20 μs, which differs from the Kelvin–Voigt case, for which the surface creeps without experiencing an immediate elastic response (see the inset in Figure 2b). The SLS parameters were: $k_e = k$ = 7.5 N/m and $c = 2.5 \times 10^{-5}$ N·s/m.

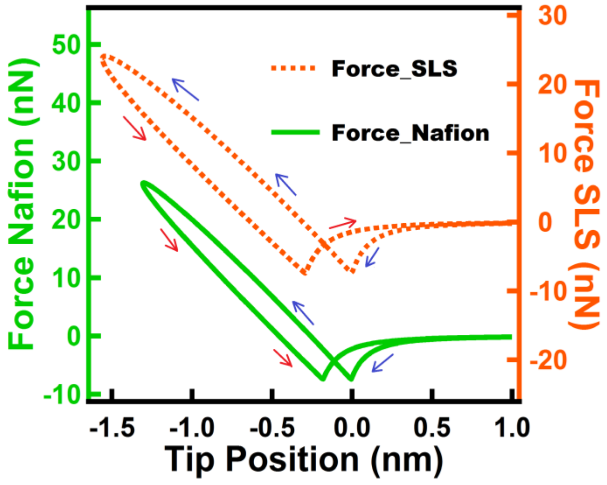

Figure S3: Typical force–distance trajectories for the SLS and the Nafion model. The cantilever dynamics parameters were: cantilever position z_c = 80 nm, natural frequency (f_0) = 25 kHz, free amplitude (A_{01}) = 100 nm, cantilever stiffness (k_{m1}) = 4 N/m. The SLS parameters were: k_e = 17.5 N/m, k = 7.5 N/m and c = 0.5 × 10^{-5} N·s/m. The Nafion parameters were: k_e = 20 N/m, k_1 = 10 N/m, k_2 = 5 N/m, c_1 = 1.0 × 10^{-5} N·s/m, c_2 = 10.0 × 10^{-5} N·s/m. The blue and red arrows correspond to approach and retraction of the tip, respectively.
